# Supplementary material for: The lipid transfer proteins Nir2 and Nir3 sustain phosphoinositide signaling and actin dynamics during phagocytosis
Source: J Cell Sci. 2023 Jul 24;136(14):jcs260902. doi: 10.1242/jcs.260902 (PMC10399989; doi:10.1242/jcs.260902)
Supplement: Supplementary information [file joces-136-260902-s1.pdf]

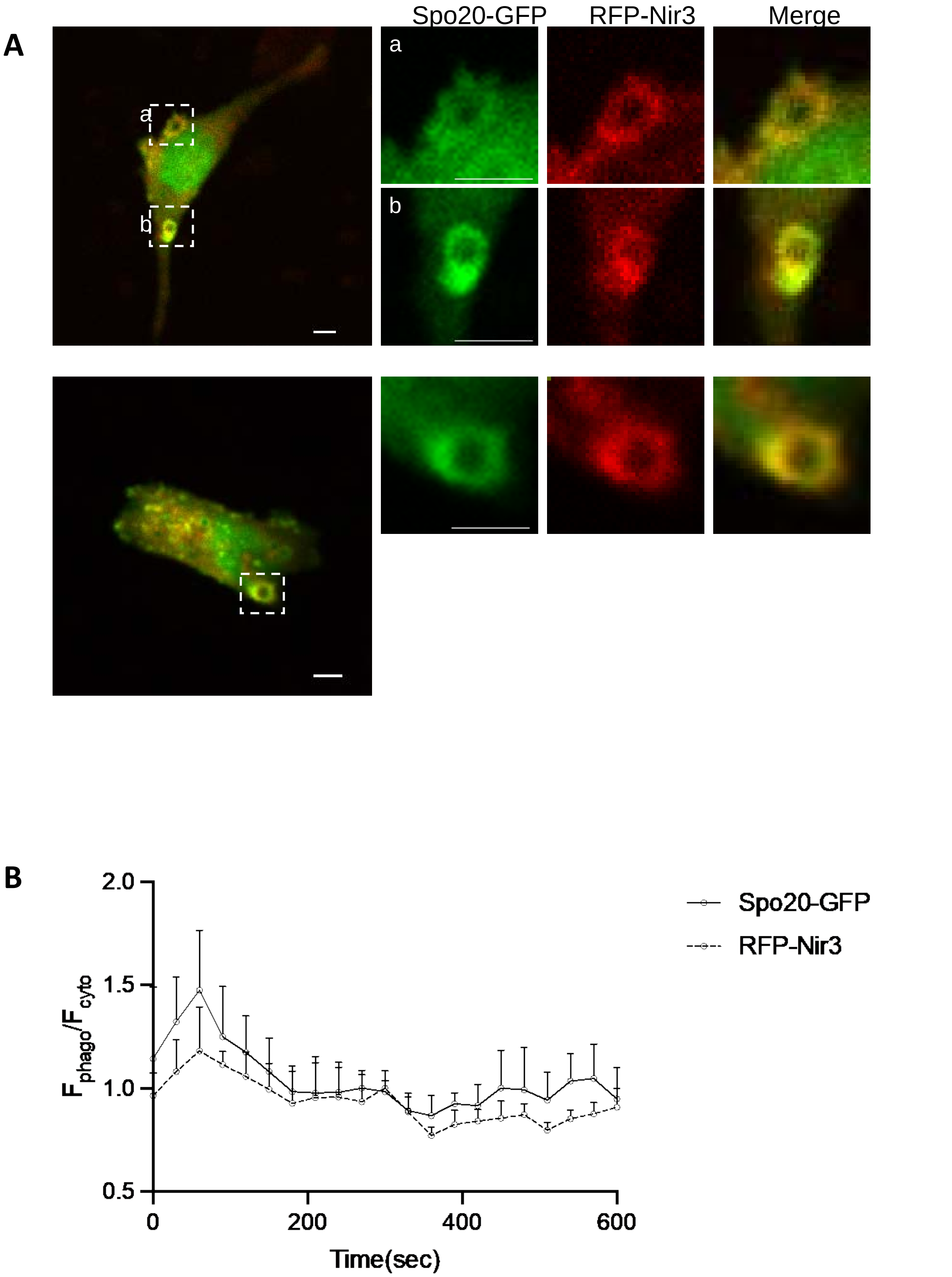

Fig. S1. Related to Fig. 1. Nir3 is recruited to phagocytic cups enriched in phosphatidic acid (PA)

A) Confocal sections of phagocytic cells co-expressing Spo20-GFP (green) and RFP-Nir3 (red) 30 min after exposure to IgG-opsonized particles. Insets show phagosome-associated fluorescence. Scale bars: 5 µm. B) Time-course of the phagosomal Spo20-GFP (solid line) and RFP-Nir3 (dashed line) increase in fluorescence during particle uptake.

Journal of Cell Science • Supplementary information

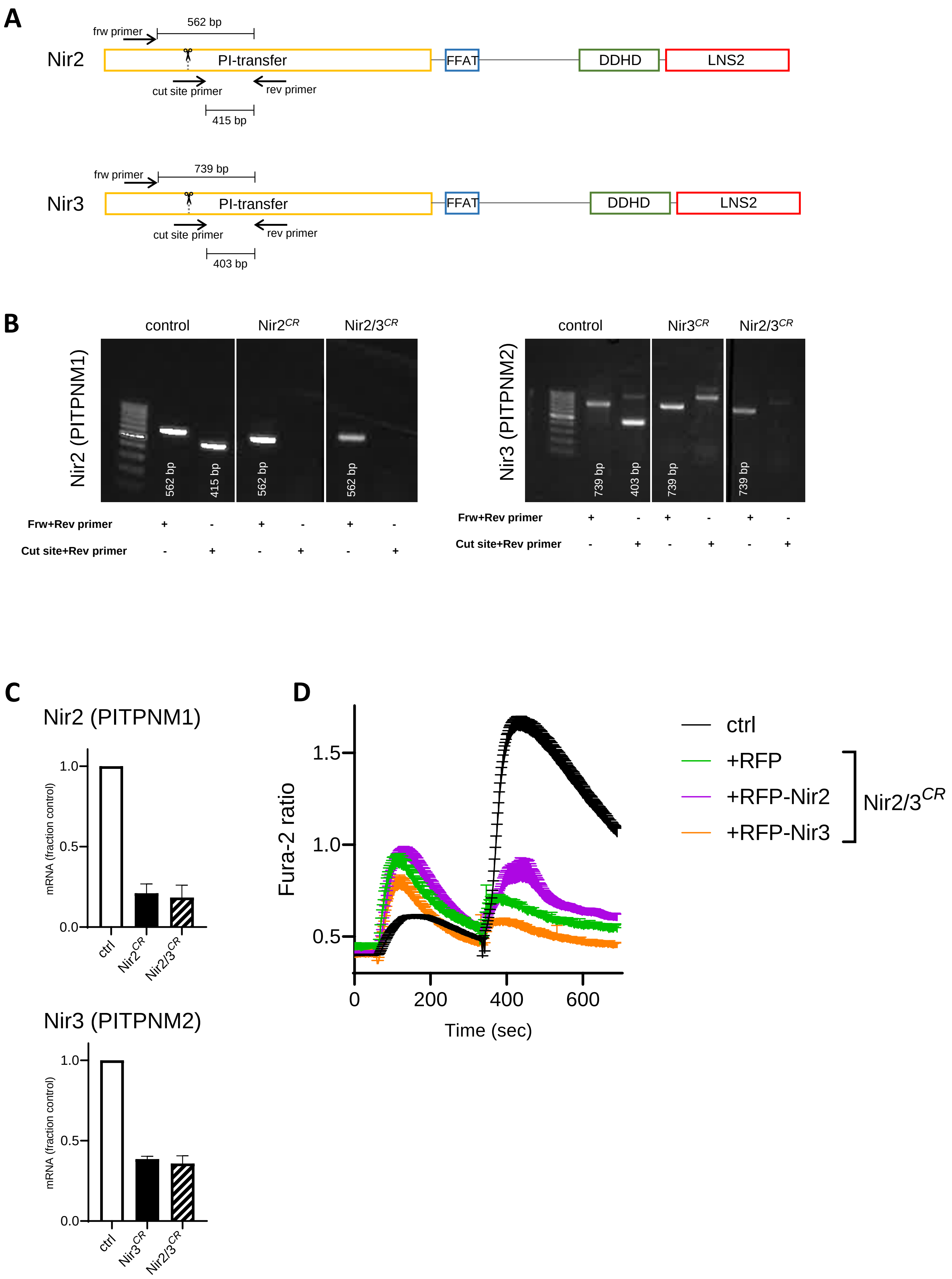

Journal of Cell Science • Supplementary information

Fig. S2. Related to Fig. 2. Genetic and functional validation of Nir2/3 depletion

A) Sites in the PITPNM genes targeted by the guide RNAs and PCR primers. B) PCR products detected by primers targeting regions outside or within the cut site in the indicated clones. C) Nir2 (top) and Nir3 (bottom) mRNA expression in the indicated clones relative to CRISPR-control cells (n=2). D) Averaged Ca^{2+} responses evoked by the addition of thapsigargin and 1 mM Ca^{2+} to Nir2/3-edited cells expressing the indicated constructs. Recordings totaling 11-35 cells.

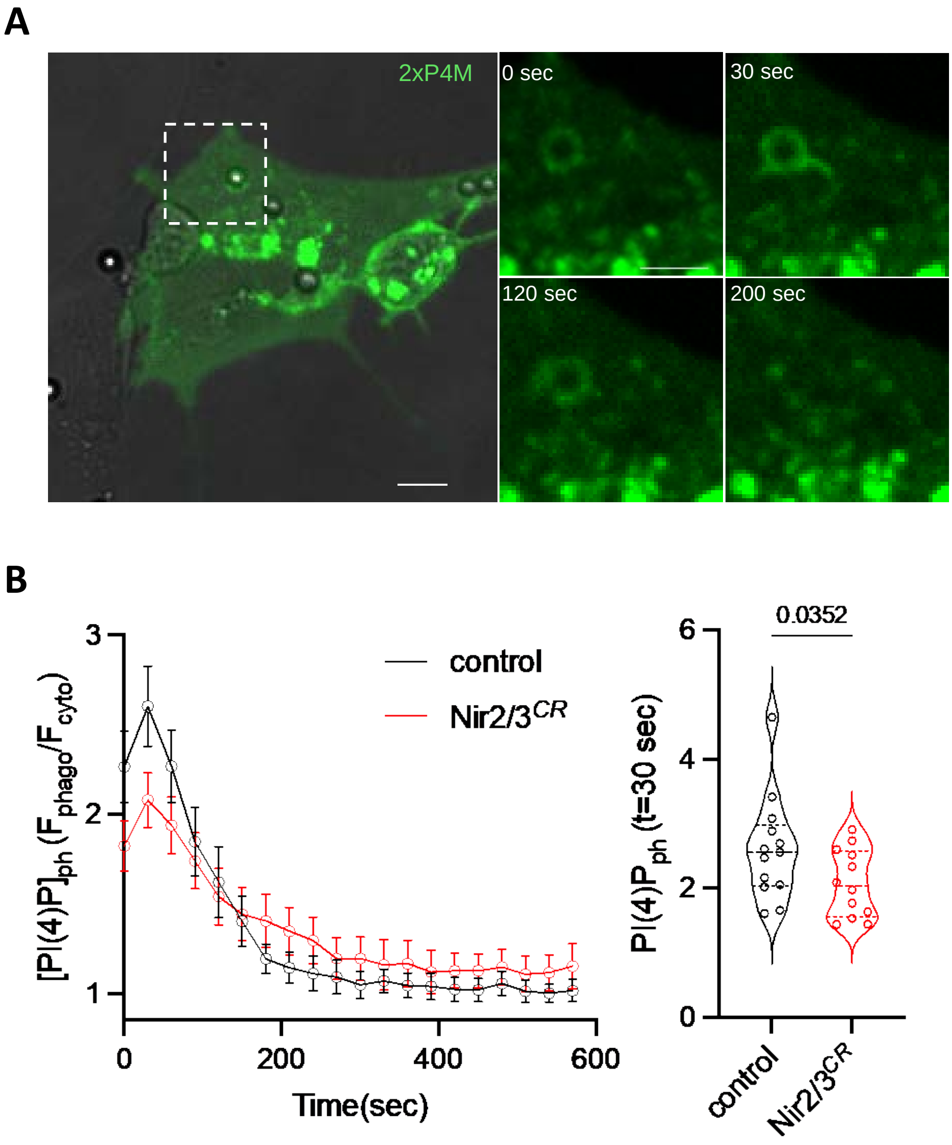

Fig. S3. Related to Fig. 4. Nir2 and Nir3 depletion reduces phagosomal PI4P enrichment

A) Time-lapse images of GFP-P4M dynamics in a control cell phagocytosing IgG-opsonized particles.

B) Left: changes in phagosomal vs. cytosolic GFP-P4M fluorescence during particle uptake by control (n=13) and Nir2-edited cells (n=12). Right: Peak GFP-P4M fluorescence increase on phagosomes 30 sec after particle capture. One-tailed unpaired t test. Scale bar: 5 µm.

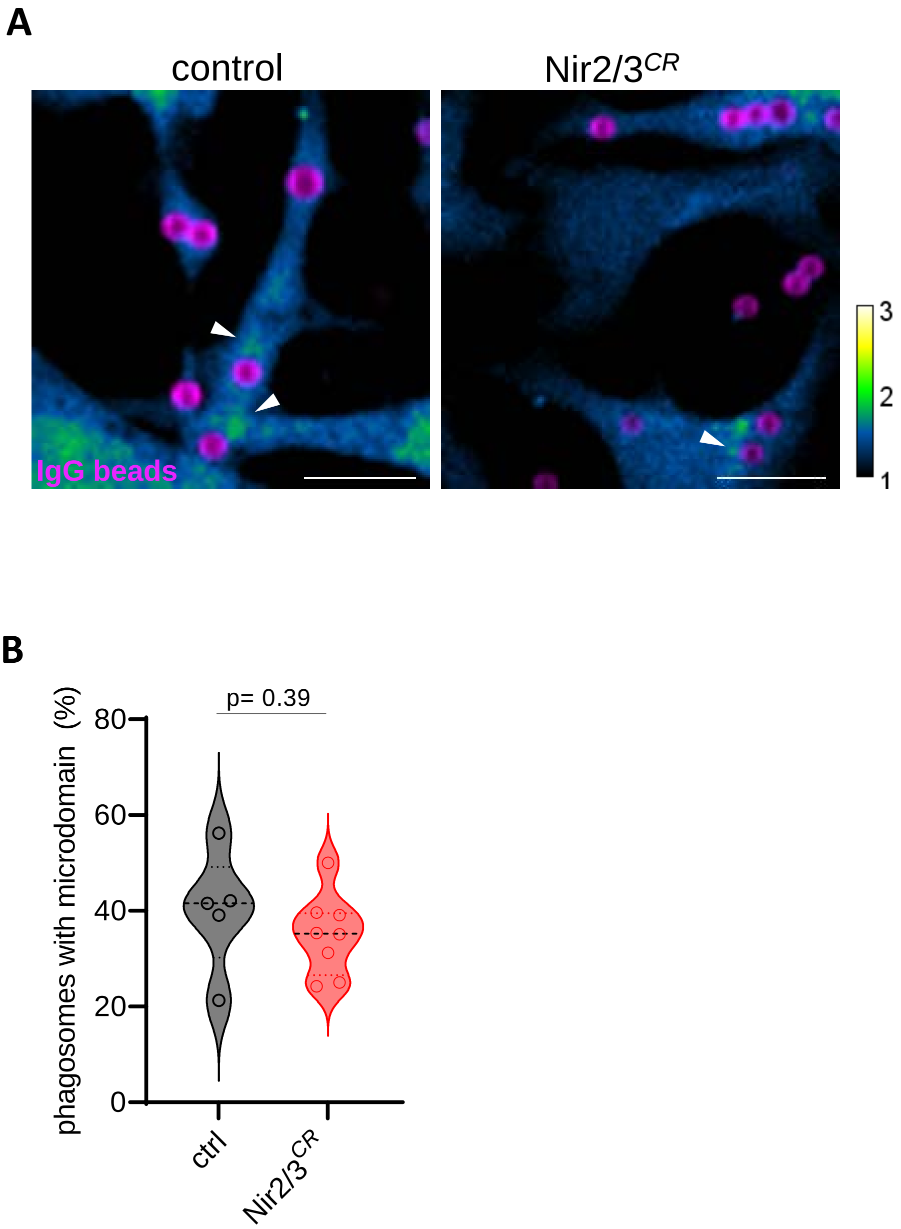

Fig. S4. Related to Fig. 4. Effect of Nir2/3 depletion on periphagosomal calcium signals

A) Fluo-8 ratio images (F/F0) of control and Nir2/3-edited cells loaded with 4 µM Fluo8-AM and 2.5µM BAPTA-AM in medium containing 2mM Ca^{2+} after 30 min of phagocytosis. Scale bars 10 µm. B) Proportion of phagosomes associated with periphagosomal Ca^{2+} hotspots in control and Nir2/3-edited cells. Two-tailed unpaired t test of n=6 and 8 recordings with 96/265 and 91/253 cells/phagosomes each for control and Nir2/3-edited cells, respectively.

Journal of Cell Science • Supplementary information

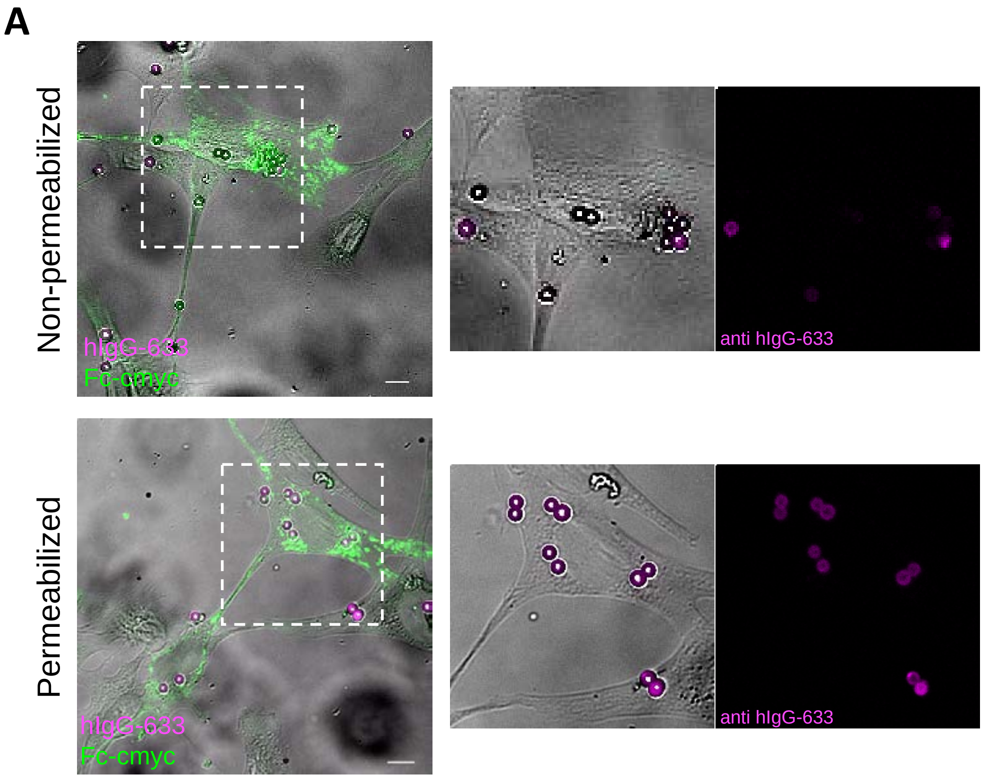

Fig. S5. Related to Fig. 4. Nir2 and Nir3 depletion stalls phagocytosis at cup stage
Bright-field and fluorescence images of cells expressing myc-FcRIIA labeled with anti-cMyc (green) and anti-hIgG (magenta) permeabilized (bottom) or not (top) with NP40 0.01% 30 min after particle exposure. Scale bars: 10 μm.

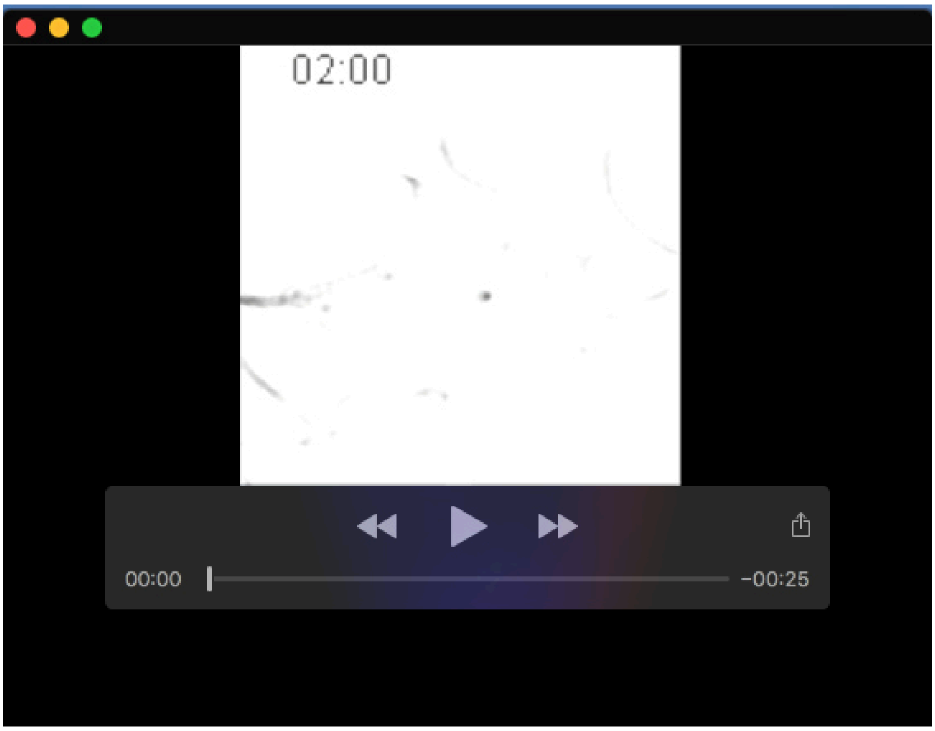

Movie 1. Related to Fig. 5. Unique actin ring formation during phagocytosis. Confocal timelapse images of LifeAct-mcherry expressing control MEF cells during phagocytosis of IgG-opsonized beads. Images were acquired 10 min after addition of IgG-opsonized targets, every 15 sec.

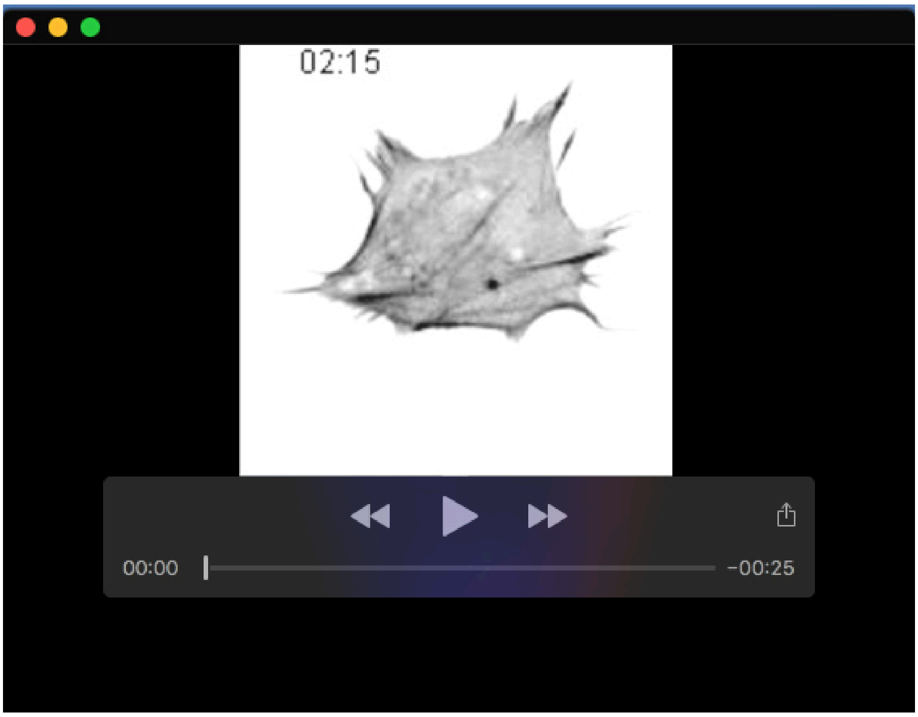

Movie 2. Related to Fig. 5. Repetitive actin ring formation during phagocytosis. Confocal timelapse images of LifeAct-mcherry expressing Nir2/3^{CR} MEF cells during phagocytosis of IgG-opsonized beads. Images were acquired 10 min after addition of IgG-opsonized targets, every 15 sec.
