# Supplementary material for: The lipid transfer proteins Nir2 and Nir3 sustain phosphoinositide signaling and actin dynamics during phagocytosis
Source: J Cell Sci. 2023 Jul 24;136(14):jcs260902. doi: 10.1242/jcs.260902 (PMC10399989; doi:10.1242/jcs.260902)
Supplement: Supplementary file 1 [file joces-136-260902_review_history.pdf]

The lipid transfer proteins Nir2 and Nir3 sustain phosphoinositide signaling and actin dynamics during phagocytosis

Mayis Kaba, Amado Carreras-Sureda, Paula Nunes-Hasler and Nicolas Demaurex
DOI: 10.1242/jcs.260902

Editor: Daniel Billadeau

Review timeline

Original submission:	21 December 2022
Editorial decision:	28 February 2023
First revision received:	30 May 2023
Editorial decision:	19 June 2023
Second revision received:	21 June 2023
Accepted:	21 June 2023

Original submission

First decision letter

MS ID#: JOCES/2022/260902

MS TITLE: The lipid transfer proteins Nir2 and Nir3 sustain phosphoinositide signaling and actin dynamics during phagocytosis

AUTHORS: Mayis Kaba, Amado Carreras-Sureda, Paula Nunes-Hasler, and Nicolas Demaurex
ARTICLE TYPE: Research Article

We have now reached a decision on the above manuscript.

To see the reviewers' reports and a copy of this decision letter, please go to: https://submit-jcs.biologists.org and click on the 'Manuscripts with Decisions' queue in the Author Area. (Corresponding author only has access to reviews.)

As you will see, the reviewers appreciated the novelty of the study but raise several concerns that prevent me from accepting the paper at this stage. They suggest, however, that a revised version might prove acceptable, if you can address their concerns. If you think that you can deal satisfactorily with the criticisms on revision, I would be pleased to see a revised manuscript. We would then return it to the reviewers.

Please ensure that you clearly highlight all changes made in the revised manuscript. Please avoid using 'Tracked changes' in Word files as these are lost in PDF conversion.

I should be grateful if you would also provide a point-by-point response detailing how you have dealt with the points raised by the reviewers in the 'Response to Reviewers' box. Please attend to all of the reviewers' comments. If you do not agree with any of their criticisms or suggestions please explain clearly why this is so.

© 2023. Published by The Company of Biologists under the terms of the Creative Commons Attribution License (https://creativecommons.org/licenses/by/4.0/).

Reviewer 1

Advance summary and potential significance to field

In this paper Kaba et al. describe the involvement of the phosphatidylinositol (PI)-phosphatidic acid (PA) exchange proteins, Nir2 and Nir3 in phagocytic membrane maturation. Phagocytic membranes display a complex pattern of lipid remodeling sequence that involves sequential changes in the levels of several species of phosphoinositides (PPIns). While these PPIns changes have been well characterized in numerous excellent publications, the question of how phagocytic membranes receive PI, the precursor lipid of PPIns, from the ER remained elusive. This study has filled this knowledge gap as it identified the Class II PI transfer proteins, Nir2 and Nir3 to be involved in the process.

The Authors described the enrichment in these PITPs of the contact zones formed between the ER and the phagocytic membranes and showed that the enrichment of the phagocytic membranes in PI(4,5)P2 is impaired in cells in which Nir2/Nir3 have been genetically eliminated. Moreover, phagocytosis was found to be impaired in such Ni2/Nir3 knockout cells yielding fewer engulfed particles. The Authors also showed that store-operated Ca2+ entry is impaired in the knockout cells, but the sustained Ca2+ elevations around the phagocytic cup were not affected. While the PI(4,5)P2 changes and the functional defects could be rescued by expression of the Nir proteins, the Ca2+ entry defect was not fully restored. Lastly, the Authors showed that the actin ring developing around the phagocytic cup is less pronounced often displaying repeated attempts of failed completion.

These are novel findings that are of great interest for the phagocytic field but also for general membrane lipid biologists.

Comments for the author

There are a few issues the addressing of which would significantly increase the value of the study:
1. Nir2 and Nir3 are recruited primarily to membrane contact sites where PA is elevated. It would greatly add to the study if the Authors could determine whether it is a PA increase in the phagocytic cup that attracts the Nir proteins or some other signal that is generated during the early phase of phagocytosis.
2. It is surprising that the Authors did not show PI4P changes, as problems in PI delivery is expected to affect PI4P more than PI(4,5)P2.
3. I also think the Authors could improve the quality of presentation of their findings. For example, the sizes of the two panels in Fig. 1C are different and while the right one is supposed to show an enlarged segment of the one shown on the left, the magnification is only slightly different. Also, scale bars are missing here (C, left and B, enlarged panels). Also, Fig. 4F should be bigger. I am not sure what S2A is supposed to demonstrate, as the localization of the expressed Nir proteins is hard to judge from these poor-quality pictures. Fig.
S2B: what is the explanation for the larger Ca2+ release observed in the knockout cells?

Minor points:
Page 3: add PMID: 23897088 to the citations describing Nir2 in maintaining PM PIP2 levels.
Page 3, below: PA generation is the main driving force for Nir2 PM attachment and not ESyt1 during signaling (PMID: 23897088 and PMID: 26028218).
Page 3 Last sentence: seems like something is missing at the end of the sentence.

Reviewer 2

Advance summary and potential significance to field

In this manuscript by Kaba et al., the authors investigated the roles of two lipid transfer proteins, Nir2 and Nir3, during phagocytosis. They found that Nir3 is enriched near ER contacts with phagocytic cups using correlative light-electron microscopy. By generating Nir2/3 depleted MEFs using CRISPR-Cas9 editing, they showed Nir2/3 are important for supporting PIP2 signals, actin

© 2023. Published by The Company of Biologists under the terms of the Creative Commons Attribution License (https://creativecommons.org/licenses/by/4.0/).

dynamics, and sealing of phagosomes during phagocytosis. Overall, the results shown in this work are solid. The findings are novel and interesting. This study provides new insights into the role of Nir2 and Nir3 in the fields of cell biology and phagocytosis.

Comments for the author

I have a few minor concerns.
1. (Pages 1 and 6) The authors claimed that “Nir-mediated lipid transfer maintains phosphoinositide homeostasis at phagocytic cups”, “Nir-mediated lipid transfer facilitates the closure of phagocytic cups” and “Nir-mediated lipid transfer facilitates the formation of contractile actin rings during phagocytic uptake”. Nevertheless, the requirement of lipid transfer activity of Nir proteins in mediating these functions was not demonstrated in this study. The authors should modify the claims (ie. change from “Nir-mediated lipid transfer” to “Nir proteins”) or perform rescue experiments using mutant Nir proteins that lack lipid transfer activity.
2. (Figure 4F) Nir2/3CR cells appeared to be in a very different shape (ie. much thinner) compared with control cells. It could be argued that this is the reason why the beads could not be fully engulfed by Nir2/3CR cells. If this is not the case, the authors should replace the images with cells of comparable shape and size.
3. (Figure 5D) The n number is low, with only 5 cells.
4. (Page 9) The authors stated in their conclusion that “Genetic disruption in the genes encoding for these proteins reduced basal PI(4,5)P2 levels in phagosomal membranes.” “basal” should be removed in this sentence since receptor activation occurs at phagosomal membranes.
5. (Page 9) The authors stated that “…aborting phagocytosis at cup stage without impacting PIP2 and calcium elevations…” This is not consistent with the findings shown in Figure 4B, revealing that PIP2 signals around phagosomes are blunted in Nir2/3 depleted cells. The conclusion should be modified to reflect their findings.
6. (Page 4) typo: EGPF should be changed to EGFP

First revision

<u>Author response to reviewers' comments</u>

DETAILED ANSWER TO REVIEWERS’ COMMENTS
The reviewers' comments are in *italics*, our answers in **bold**. The page numbers refer to the revised manuscript with changes highlighted in track mode

Reviewer 1 Advance Summary and Potential Significance to Field:
In this paper Kaba et al. describe the involvement of the phosphatidylinositol (PI)-phosphatidic acid (PA) exchange proteins, Nir2 and Nir3 in phagocytic membrane maturation. Phagocytic membranes display a complex pattern of lipid remodeling sequence that involves sequential changes in the levels of several species of phosphoinositides (PPIns). While these PPIns changes have been well characterized in numerous excellent publications, the question of how phagocytic membranes receive PI, the precursor lipid of PPIns, from the ER remained elusive. This study has filled this knowledge gap as it identified the Class II PI transfer proteins, Nir2 and Nir3 to be involved in the process.

The Authors described the enrichment in these PITPs of the contact zones formed between the ER and the phagocytic membranes and showed that the enrichment of the phagocytic membranes in PI(4,5)P2 is impaired in cells in which Nir2/Nir3 have been genetically eliminated. Moreover, phagocytosis was found to be impaired in such Ni2/Nir3 knockout cells yielding fewer engulfed particles. The Authors also showed that store-operated Ca2+ entry is impaired in the knockout cells, but the sustained Ca2+ elevations around the phagocytic cup were not affected. While the PI(4,5)P2 changes and the functional defects could be rescued by expression of the Nir proteins, the Ca2+ entry defect was not fully restored. Lastly, the Authors showed that the actin ring developing around the phagocytic cup is less pronounced often displaying repeated attempts of

© 2023. Published by The Company of Biologists under the terms of the Creative Commons Attribution License (https://creativecommons.org/licenses/by/4.0/).

failed completion.

These are novel findings that are of great interest for the phagocytic field but also for general membrane lipid biologists.

We thank the reviewer for the careful evaluation of our study. As suggested, we have performed additional experiments to enhance the value of our work.

Reviewer 1 Comments for the Author:
There are a few issues the addressing of which would significantly increase the value of the study:

1. Nir2 and Nir3 are recruited primarily to membrane contact sites where PA is elevated. It would greatly add to the study if the Authors could determine whether it is a PA increase in the phagocytic cup that attracts the Nir proteins or some other signal that is generated during the early phase of phagocytosis.

To address this point, we have measured the accumulation of phosphatidic acid around phagosomes using GFP fused to the membrane-binding motif of the yeast SNARE protein Spo20. These new data (Fig. S1) show that Nir3 accumulates around phagosomes enriched in PA, and that the peak of PA accumulation coincides with Nir3 recruitment to closing phagosomes. We thank the reviewer for suggesting this experiment.

2.It is surprising that the Authors did not show PI4P changes, as problems in PI delivery is expected to affect PI4P more than PI(4,5)P2.

We have measured the accumulation of PI4P around phagosomes using GFP fused to the P4M domain of SidM (GFP-P4M). These new data (Fig. S4A) show that Nir2/3 depletion reduces the transient accumulation of PI4P around forming phagosomes, but not the steady-state levels of the lipid on internalized phagosomes. Therefore, Nir2/3 editing differentially impacts PI4P and PI(4,5) P2 dynamics, minimizing acute changes in PI4P and decreasing the overall PI45P2 levels during phagocytosis. We thank the reviewer for suggesting this experiment.

3. I also think the Authors could improve the quality of presentation of their findings. For example, the sizes of the two panels in Fig. 1C are different and while the right one is supposed to show an enlarged segment of the one shown on the left, the magnification is only slightly different. Also, scale bars are missing here (C, left and B, enlarged panels). Also, Fig. 4F should be bigger. I am not sure what S2A is supposed to demonstrate, as the localization of the expressed Nir proteins is hard to judge from these poor-quality pictures. Fig. S2B: what is the explanation for the larger Ca2+ release observed in the knockout cells?

We have equalized the sizes and enlarged the magnification of the zoomed-in panels in Fig 1C, added the missing size bars in Fig.1B-C, enlarged panel 4F, and removed Fig. S2A which we agree was not very informative. In Fig. S2B (now revised Fig S2D) the Ca^{2+} release was not larger in knockout cells but reduced in non-edited cells (compare the black traces in Fig 2C and Fig S2B). The amount of Ca^{2+} released from the ER varied between different clones of WT cells but was on average comparable to the amount of Ca^{2+} mobilized by Tg in Nir-edited cells. We acknowledge that the "control" label on the black trace in Fig. S2D is misleading as this trace merely illustrates the magnitude of normal SOCE response. The appropriate control condition here is the Nir-edited cells expressing cytosolic RFP. Comparison of the colored traces show that expression of RFP-Nir2 or RFP-Nir3 does not restore SOCE in this cellular background. We have relabeled the black trace "WT" to indicate that these cells have a different genetic background. We thank the reviewer for pointing this out and for the suggestions to improve the quality of presentation of our findings.

Minor points:

Page 3: add PMID: 23897088 to the citations describing Nir2 in maintaining PM PIP2 levels.
Done

© 2023. Published by The Company of Biologists under the terms of the Creative Commons Attribution License (https://creativecommons.org/licenses/by/4.0/).

Page 3, below: PA generation is the main driving force for Nir2 PM attachment and not ESyt1 during signaling (PMID: 23897088 and PMID: 26028218).
Mentioned, thank you.

Page 3 Last sentence: seems like something is missing at the end of the sentence.
Corrected.

Reviewer 2 Advance Summary and Potential Significance to Field:

In this manuscript by Kaba et al., the authors investigated the roles of two lipid transfer proteins, Nir2 and Nir3, during phagocytosis. They found that Nir3 is enriched near ER contacts with phagocytic cups using correlative light-electron microscopy. By generating Nir2/3 depleted MEFs using CRISPR-Cas9 editing, they showed Nir2/3 are important for supporting PIP2 signals, actin dynamics, and sealing of phagosomes during phagocytosis. Overall, the results shown in this work are solid. The findings are novel and interesting. This study provides new insights into the role of Nir2 and Nir3 in the fields of cell biology and phagocytosis.

We thank the reviewer for the thorough evaluation of our study.

Reviewer 2 Comments for the Author:

I have a few minor concerns.

1.(Pages 1 and 6) The authors claimed that "Nir-mediated lipid transfer maintains phosphoinositide homeostasis at phagocytic cups", "Nir-mediated lipid transfer facilitates the closure of phagocytic cups" and "Nir-mediated lipid transfer facilitates the formation of contractile actin rings during phagocytic uptake". Nevertheless, the requirement of lipid transfer activity of Nir proteins in mediating these functions was not demonstrated in this study. The authors should modify the claims (ie. change from "Nir-mediated lipid transfer" to "Nir proteins") or perform rescue experiments using mutant Nir proteins that lack lipid transfer activity.

We have attempted to re-express Nir proteins truncated or bearing point mutation within the PI transfer domain but could not obtain enough expressing cells using transient transfection procedures to accurately quantify phagocytosis. Addressing this point would require the generation of clones stably re-expressing the mutated proteins, which would delay excessively the publication of the present study. We have therefore as suggested removed the statements referring to the lipid transfer activity of Nir proteins throughout the manuscript.

2.(Figure 4F) Nir2/3CR cells appeared to be in a very different shape (ie. much thinner) compared with control cells. It could be argued that this is the reason why the beads could not be fully engulfed by Nir2/3CR cells. If this is not the case, the authors should replace the images with cells of comparable shape and size.

We did not observe major morphological difference between control and Ni2/3 edited cells by live confocal imaging. Cells also appeared comparable in shape and size on TEM sections as illustrated on the image below. Phagocytic cups are preferentially found in the cell periphery and we randomly selected phagocytic events on TEM sections regardless of their cellular location. The simplest explanation for the thinner aspect of Nir2/3CR cells on TEM sections is thus that our sampling reflects the preferential location of cups.

© 2023. Published by The Company of Biologists under the terms of the Creative Commons Attribution License (https://creativecommons.org/licenses/by/4.0/).

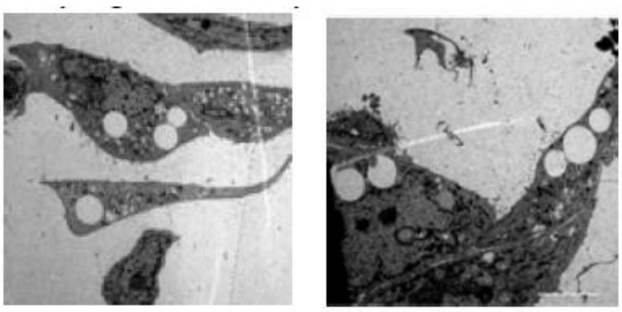

***Figure R1: Electron micrographs of control and Nir2/3-edited cells 30 min after particle exposure**. Bar 5 µm*

3.(Figure 5D) The n number is low, with only 5 cells.

We have reanalyzed the entire dataset using an automated image segmentation procedure to quantify the presence of mCherry-actin within a 1 µM-wide annular region around phagosomes. The new Fig. 5C-D shows that this periphagosomal region is almost entirely covered by actin in control cells and that this recruitment is prevented by Nir2/3 depletion (N=14-20).

4.(Page 9) The authors stated in their conclusion that "Genetic disruption in the genes encoding for these proteins reduced basal PI(4,5)P2 levels in phagosomal membranes." "basal" should be removed in this sentence since receptor activation occurs at phagosomal membranes. **Corrected, thank you.**

5.(Page 9) The authors stated that "…aborting phagocytosis at cup stage without impacting PIP2 and calcium elevations…" This is not consistent with the findings shown in Figure 4B, revealing that PIP2 signals around phagosomes are blunted in Nir2/3 depleted cells. The conclusion should be modified to reflect their findings.

We have modified the sentence as suggested, from "aborting phagocytosis at cup stage without impacting PIP2 and calcium elevations" to "decreasing PM and phagosomal PIP2 levels and aborting phagocytosis at cup stage without impacting calcium elevations".

6.(Page 4) typo: EGPF should be changed to EGFP **Done**

Second decision letter

MS ID#: JOCES/2022/260902

MS TITLE: The lipid transfer proteins Nir2 and Nir3 sustain phosphoinositide signaling and actin dynamics during phagocytosis

AUTHORS: Mayis Kaba, Amado Carreras-Sureda, Paula Nunes-Hasler, and Nicolas Demaurex
ARTICLE TYPE: Research Article

We have now reached a decision on the above manuscript.

To see the reviewers' reports and a copy of this decision letter, please go to: https://submit-jcs.biologists.org and click on the 'Manuscripts with Decisions' queue in the Author Area. (Corresponding author only has access to reviews.)

As you will see, Reviewer 2 has requested two edits to the text. Please take care of these and return the revision promptly.

© 2023. Published by The Company of Biologists under the terms of the Creative Commons Attribution License (https://creativecommons.org/licenses/by/4.0/).

Please ensure that you clearly highlight all changes made in the revised manuscript. Please avoid using 'Tracked changes' in Word files as these are lost in PDF conversion.

I should be grateful if you would also provide a point-by-point response detailing how you have dealt with the points raised by the reviewers in the 'Response to Reviewers' box. Please attend to all of the reviewers' comments. If you do not agree with any of their criticisms or suggestions please explain clearly why this is so.

Reviewer 1

Advance summary and potential significance to field

In this revised paper Kaba et al. describe the involvement of the phosphatidylinositol (PI)-phosphatidic acid (PA) exchange proteins, Nir2 and Nir3 in phagocytic membrane maturation. Phagocytic membranes display a complex pattern of lipid remodeling sequence that involves sequential changes in the levels of several species of phosphoinositides (PPIns). While these PPIns changes have been well characterized in numerous excellent publications, the question of how phagocytic membranes receive PI, the precursor lipid of PPIns, from the ER remained elusive. This study has filled this knowledge gap as it identified the Class II PI transfer proteins, Nir2 and Nir3 to be involved in the process.
In the revised manuscript the Authors have addressed all of my previous comments and added new experimental data. The revised manuscript has substantially improved.

Comments for the author

None

Reviewer 2

Advance summary and potential significance to field

My previous comments have been sufficiently addressed in the revised manuscript. Nevertheless, there are two minor comments that should be addressed before publication.

Comments for the author

Page 1 (Abstract)
The author stated "Nir3 and to a lesser extent Nir2 accumulated in ER cisternae juxtaposed to phagocytic cups when expressed in phagocytic mouse fibroblasts". However, the experiments that showed recruitment of Nir2 and Nir3 to ER-phagosome contact sites were done in Cos7 cells instead of mouse fibroblasts. The statement should be modified accordingly. In addition, it is confusing to state that cytosolic Nir2 and Nir3 accumulate "in" ER cisternae.

Page 3 (Introduction)

During revision, the author added a new citation (PMID 23897088) in the introduction to support the statement "Nir2 maintains PI(4,5)P2 levels at the PM during signaling of Gq-coupled receptors.....". The author should also cite Chang et al. 2013 (PMID 24183667), which was the first to show that Nir2 maintains PM PIP2 following Gq-coupled receptor activation and that Nir2 localizes at ER membrane contact sites in receptor-stimulated cells.

© 2023. Published by The Company of Biologists under the terms of the Creative Commons Attribution License (https://creativecommons.org/licenses/by/4.0/).

Second revision

Author response to reviewers' comments

Dear Reviewers,

Thank you very much for your positive appreciation of our manuscript.

As suggested by reviewer 2 we have edited our abstract to specify that COS-7 cells were used to track the recruitment of expressed proteins by confocal microscopy and CLEM . We have also added the Chang et al (2013) paper in the introduction to acknowledge the discovery of Nir2-mediated lipid transfer during signaling at ER-PM contact sites. The changes are highlighted in bold red in the "text with changes highlighted" file.

We appreciate your time and effort in evaluating this manuscript which we hope is now suitable for publication in Journal of Cell Science.

Sincerely yours,

Nicolas Demaurex (on behalf of all the authors)

Third decision letter

MS ID#: JOCES/2022/260902

MS TITLE: The lipid transfer proteins Nir2 and Nir3 sustain phosphoinositide signaling and actin dynamics during phagocytosis

AUTHORS: Mayis Kaba, Amado Carreras-Sureda, Paula Nunes-Hasler, and Nicolas Demaurex
ARTICLE TYPE: Research Article

I am happy to tell you that your manuscript has been accepted for publication in Journal of Cell Science, pending standard ethics checks.

© 2023. Published by The Company of Biologists under the terms of the Creative Commons Attribution License (https://creativecommons.org/licenses/by/4.0/).
